# Draft Genome Sequence of the Fast-Growing Marine Bacterium *Vibrio natriegens* Strain ATCC 14048

**DOI:** 10.1128/genomeA.00589-13

**Published:** 2013-08-08

**Authors:** Zheng Wang, Baochuan Lin, W. Judson Hervey, Gary J. Vora

**Affiliations:** Center for Bio/Molecular Science and Engineering, Naval Research Laboratory, Washington, DC, USA

## Abstract

*Vibrio natriegens* bacteria are Gram-negative aquatic microorganisms that are found primarily in coastal seawater and sediments and are perhaps best known for their high growth rates (generation time of <10 min). In this study, we report the first sequenced genome of this species, that of the type strain *Vibrio natriegens* ATCC 14048, a salt marsh mud isolate from Sapelo Island, GA.

## GENOME ANNOUNCEMENT

*Vibrio natriegens* (originally described as *Pseudomonas natriegens* [1] and then *Beneckea natriegens* [2]) is a moderately halophilic member of the Harveyi clade (3) and *Vibrio* core group (4) that is commonly found in marine and estuarine coastal waters and sediments. Members of the *natriegens* species in general are tremendously versatile organoheterotrophs, as they exhibit the ability to utilize a diverse variety of organic substrates as carbon and energy sources (2). *V. natriegens* is also recognized as one of the fastest-dividing organisms currently known, with a documented doubling time of 9.8 min (5). This is a growth rate that requires an extremely high rate of protein synthesis, which is accommodated by a higher rRNA gene dose, increases in ribosome numbers, strong rRNA promoters (6), and, potentially, codon usage bias (7). Its nutritional versatility, rapid doubling time, and the fact that it is nonpathogenic to humans have all encouraged the use of this species in teaching exercises and physiological studies (8, 9). These characteristics also suggest that if better understood, *V. natriegens* could become a valuable source for genetic parts or a novel biological chassis capable of rapid biosyntheses for synthetic biological applications (10).

To begin to investigate this potential, we sequenced the genome of the *V. natriegens* type strain ATCC 14048 (NBRC 15636, DSM 759) using an Illumina MiSeq benchtop sequencer. The read library (~300-bp inserts) was composed of 15,999,016 2- × 250-bp paired-end reads that resulted in 779× coverage. Assembly of the reads using the Ray *de novo* assembly software (11) with a k-mer value of 49 produced 39 scaffolds (>500 bp), and a genome-scale assembly was constructed with Mauve genome alignment software (version 2.3.1; http://asap.ahabs.wisc.edu) using the closest fully sequenced relative of *V. natriegens*, *Vibrio* sp. strain EJY3 (12), as a reference scaffold. Gene prediction and annotation were performed using the RAST (Rapid Annotation using Subsystem Technology) server (13) and the NCBI Prokaryotic Genome Automatic Annotation Pipeline (PGAAP).

The assembled draft genome of *V. natriegens* ATCC 14048 is 5,131,685 bp in size with 4,587 RAST-server-annotated open reading frames contained within two circular chromosomes. The 3,202,568-bp chromosome I (43.7% G+C; 86.1% coding sequences, 13.3% hypothetical) is composed of 27 supercontigs and contains 11 rRNA operons and at least 103 tRNAs. In comparison, the 1,929,117-bp chromosome II (42.1% G+C; 85.2% coding sequences, 19.5% hypothetical) is composed of 12 supercontigs and contains 1 rRNA operon and at least 21 tRNAs. The genome also harbored seven insertion sequence elements but no retrons. Further analyses are now under way to better elucidate the genetics of *V. natriegens* and to develop tools to potentially exploit this organism as a platform for rapid biosynthesis.

### Nucleotide sequence accession numbers.

This whole-genome shotgun project has been deposited at DDBJ/EMBL/GenBank under the accession number ATFJ00000000. The version described in this paper is version ATFJ01000000.
